# Looking for the best anti-colitis medicine: A comparative analysis of current and prospective compounds

**DOI:** 10.18632/oncotarget.13894

**Published:** 2016-12-10

**Authors:** Anastasiya A. Chumanevich, Anusha Chaparala, Erin E. Witalison, Hossam Tashkandi, Anne B. Hofseth, Corey Lane, Edsel Pena, Piaomu Liu, Doug L. Pittman, Prakash Nagarkatti, Mitzi Nagarkatti, Lorne J. Hofseth, Alexander A. Chumanevich

**Affiliations:** ^1^ Department of Drug Discovery and Biomedical Sciences, South Carolina College of Pharmacy, University of South Carolina, SC, USA; ^2^ Department of Statistics, University of South Carolina, Columbia, SC, USA; ^3^ School of Medicine, University of South Carolina, Columbia, SC, USA

**Keywords:** colitis, colon, inflammation, dextran sulfate sodium, CAM, FDA

## Abstract

Ulcerative colitis (UC) is a chronic lifelong inflammatory disorder of the colon, which, while untreated, has a relapsing and remitting course with increasing risk of progression toward colorectal cancer. Current medical treatment strategies of UC mostly focus on inhibition of the signs and symptoms of UC to induce remission and prevent relapse of disease activity, minimizing the impact on quality of life, but not affecting the cause of disease. To date, however, there is no single reliable treatment agent and/or strategy capable of effectively controlling colitis progression throughout the patient's life without side effects, remission, or resistance. Taking into consideration an urgent need for the new colitis treatment strategies, targets and/or modulators of inflammation, we have tested current and prospective compounds for colitis treatment and directly compared their anti-colitis potency using a dextran sulfate sodium (DSS) mouse model of colitis. We have introduced a composite score – a multi-parameters comparison tool – to assess biological potency of different compounds.

## INTRODUCTION

Correlated with inflammatory bowel disease (IBD), ulcerative colitis and Crohn's disease are characterized as two prevailing chronic illnesses with obscure causing agents, conditions, and perennial damage. Autoimmune gastrointestinal disorders heighten colon cancer risk with progressing severity through longer duration of colitis, greater anatomic colitis extent, and other/additional potentially contributing carcinogenic inflammatory pathways [1]. In ulcerative colitis patients, the risk of occurrence of colitis-associated colorectal cancer increases with age [2, 3].

Due to advances in the understanding of IBD in the past two decades, several FDA-approved drugs are currently available for colitis treatment.

For the mild form of colitis, 5-Aminosalicylic acid (5-ASA) and its derivatives (e.g. sulfasalazine and mesalamine) have been the standard treatment, and may play a chemopreventive role against colitis-driven colon cancer [4]. However, the response rates to these drugs usually are in the range of 40-60%, and are never 100% [5]. Moreover, there are serious side-effects associated with 5-ASA treatment of colitis, including hepatitis, pancreatitis, and pulmonary dysfunction [6], and in some cases, these agents can even increase the colitis severity [5].

For moderate-to-severe forms of colitis, immunosuppressive agents (mainly against TNFα) are widely used, and have become the standard of care. However, this aggressive strategy has extremely dangerous side effects, including a risk for non-Hodgkins lymphoma [7], and an increased risk of infection, especially tuberculosis and reactivation of viral hepatitis [8–10]. Other immune modulators used in the clinic include Azathioprine and mercaptopurine, but these work extremely slowly (i.e. up to three months before clinical effects take place), and also include side effects, such as allergic reactions, infections, hepatic and pancreatic inflammation, and cancer [9, 11].

Another anti-colitis agent, Cyclosporine A, which is normally reserved for patients who do not adequately respond to other medications, begins working in one to two weeks, but with severe side effects, including kidney damage and fatal infections [9, 12].

Corticosteroids can help reduce inflammation, but also possess side effects such as weight gain, facial hair in men and women, high blood pressure, type 2 diabetes, osteoporosis, and susceptibility to infections [9].

Thus, there is no current reliable treatment strategy for colitis and the prevention of colon cancer associated with colitis (especially moderate to severe) that lacks side effects, completely inhibits the signs and symptoms of disease pathology, and overcomes patients' eventual resistance to many treatment agents. For these reasons, new treatment strategies, targets, and/or new modulators of inflammation with none or minimal toxicity are necessary to battle colitis and prevent colon cancer.

Our lab has a long history of studying natural complementary and alternative medicine (CAM) compounds, as well as synthetic small molecule compounds to battle colitis and colon cancer associated with colitis. We have shown that the crude extract from the plant American Ginseng (AG), and a product of its further fractionation – a Hexane fraction of AG (HAG)– effectively suppresses colitis and colon cancer through apoptosis of inflammatory cells and cell cycle arrest of colon cancer cells, involving preferential suppression of STAT/iNOS signaling in activated macrophages [13], prevention of DNA damage in colon cells [14], and prevention of colon cancer cell migration through enhanced miR-29b expression [15]. Similarly, resveratrol, another naturally occurring compound, is an effective suppressor of tumor-promoting inflammation [16–18], most likely by enhancing the expression of silent mating type information regulation (SIRT-1) and subsequent downregulation of NF-κB, which plays a crucial role in colitis and colon cancer associated with colitis [19].

Recently we have identified and described several synthetic small molecule agents that effectively battle colitis in mice. Among these – Cl-Amidine [20, 21] and its derivative BB-Cl-Amidine [22, 23]. Both of these compounds inhibit the protein arginine deiminase (PAD) family of enzymes. Dysregulated activity of these enzymes plays a substantial role in the onset and progression of multiple human chronic conditions, including colitis and colon cancer [24–28]. In our previous studies, we have shown that these PAD inhibitors effectively suppress ulcerative colitis in the dextran sulfate sodium (DSS) mouse model, and can be effectively used to prevent and treat this high colon cancer risk disease [29–32].

Our latest advance in the treatment of IBD involves the use of the FDA-approved anti-malarial drug, quinacrine. Quinacrine has a long history of successful applications for the treatment of malaria, tape-worm infections, giardiasis and systemic lupus erythematosus, possesses antioxidant properties, and has efficacy against autoimmune disorders, such as rheumatoid arthritis with rare, but mild side effects [33–39]. We have shown that quinacrine successfully suppresses colitis without any indication of toxicity or side effects in two mouse models of UC [40].

In this study, we have tested six compounds, including CAMs and synthetic drugs known to possess anti-colitis properties, to directly compare their anti-colitis potency versus two current FDA-approved treatments, in three doses each, using a DSS mouse model of colitis.

## RESULTS

### Suppression of colitis in a DSS mouse model of colitis in a dose-dependent manner

**Figure 1:**
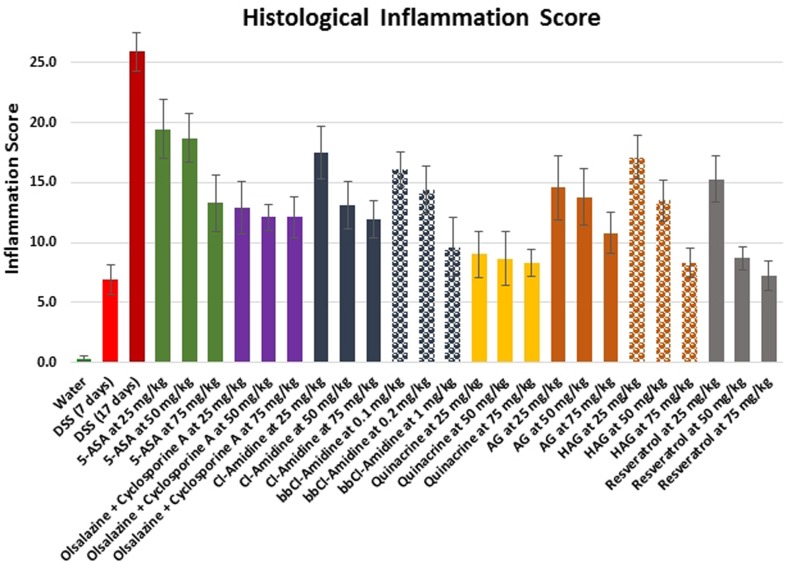
Effects of the treatment on histological inflammation score in the DSS model of colitis Values represent mean ± S.E. The differences between experimental groups and 17 days DSS only group were statistically significant for all groups (*p* < 0.05).

UC is associated with chronic inflammation in the bowels. We tested all compounds on mice to directly compare their efficiency to suppress DSS-induced acute colitis based on histological inflammation scoring. The histological inflammation score was determined from the H&E-stained colon sections of each mouse treated with test compounds during 17 days of exposure to DSS, taking into consideration the inflammation severity and extent, ulceration areas, as well as pathological crypt changes. Supplemental Figure 1 contains representative images, demonstrating our approach. The comparative results are shown in Figure 1 and in Table 1.

**Table 1:** Gross characteristics of treated groups

Treatment Groups	Starting Weight, g	Ending Weight, g	Weight Difference, g	Colon Length, cm	Inflammation Score	RBC, b/mm^3^ ^††^	WBC, m/mm^3^ ^†^	Lymphocytes, m/mm^3^ ^†^
Water	24.4 ± 0. 46	26.2 ± 0.60	1.8 ± 0.27	8.8 ± 0.19	0.3 ± 0.21	10.4 ± 0.16	8.9 ± 0.41	7.8 ± 0.41
DSS (7 days)	24.1 ± 0.79	24.1± 0.80	−0.03 ± 0.14	7.9 ± 0.16	6.9 ± 1.22	10.6 ± 0.25	10.0 ± 0.52	8.6 ± 0.41
DSS (17 days)	23.9 ± 0.51	22.3 ± 0.60	−1.6 ± 0.56	6.9 ± 0.22	25.9 ± 1.6	9.5 ± 0.11	10.2 ± 0.47	8.2 ± 0.36
5-ASA at 25 mg/kg	24.9 ± 0.49	23.2 ± 0.68	−1.6 ± 0.52	7.4 ± 0.13	19.4 ± 2.46	9.9 ± 0.29	5.2 ± 0.60	3.6 ± 0.52
5-ASA at 50 mg/kg	25.6 ± 0.42	24.5 ± 0.43	−1.1 ± 0.3	7.5 ± 0.18	18.7 ± 2.03	9.8 ± 0.33	6.4 ± 0.35	3.9 ±0.25
5-ASA at 75 mg/kg	22.6 ± 0.73	21.8 ± 0.39	−0.8 ± 0.57	7.6 ± 0.23	13.3 ± 2.36	10.5 ± 0.13	6.4 ± 0.63	4.5 ± 0.43
Olsalazine + Cyclosporine A at 25 mg/kg	23.7 ± 0.54	23.8 ± 0.63	0.1 ± 0.17	7.8 ± 0.12	12.9 ± 2.18	9.9 ± 0.22	7.5 ± 0.44	6.1 ± 0.32
Olsalazine + Cyclosporine A at 50 mg/kg	24.4 ± 0.33	25 ± 0.39	0.6 ± 0.22	8.1 ± 0.25	12.1 ± 1.1	10.3 ± 0.16	7.6 ± 0.54	6.4 ± 0.41
Olsalazine + Cyclosporine A at 75 mg/kg	22.4 ±0.35	22.8 ± 0.25	0.4 ± 0.19	8.1 ± 0.23	12.1 ± 1.68	10.4 ± 0.14	6.0 ± 0.41	5.0 ± 0.39
Cl-Amidine at 25 mg/kg	25.4 ± 0.57	26 ± 0.70	0.7 ± 0.55	7.9 ± 0.17	17.5 ± 2.18	9.2 ± 0.23	7.9 ± 0.52	6.3 ± 0.46
Cl-Amidine at 50 mg/kg	24.6 ± 0.46	26 ± 0.46	1.4 ± 0.33	8.5 ± 0.27	13.1 ± 2	9.4 ± 0.27	7.5 ± 0.38	6.3 ± 0.35
Cl-Amidine at 75 mg/kg	25.0 ± 0.74	26.1 ± 0.60	1.1 ± 0.37	8.3 ± 0.14	11.9 ± 1.54	9.5 ± 0.28	8.1 ± 0.55	6.5 ± 0.41
BB-Cl-Amidine at 0.1 mg/kg	24.3 ± 0.38	23.7 ± 0.34	−0.6 ± 0.39	8.2 ± 0.30	16.1 ± 1.40	9.1 ± 0.19	7.6 ± 1.00	6.2 ± 0.63
BB-Cl-Amidine at 0.2 mg/kg	25.0 ± 0.80	24.6 ± 0.64	−0.4 ± 0.25	8.1 ± 0.17	14.4 ± 1.96	8.3 ± 0.32	8.1 ± 0.95	6.4 ± 0.72
BB-Cl-Amidine at 1 mg/kg	25.1 ± 0.38	26.4 ± 0.56	1.3 ± 0.35	7.5 ± 0.33	9.6 ± 0.30	9.0 ± 0.68	8.6 ± 1.05	7.2 ± 0.87
Quinacrine at 25 mg/kg	25.3 ± 0.32	24.7 ± 0.37	−0.7 ± 0.38	8.1 ± 0.15	9 ± 1.97	10.0 ± 0.32	6.4 ± 0.0.47	4.5 ± 0.39
Quinacrine at 50 mg/kg	24.8 ± 0.46	23.4 ± 0.88	−0.9 ± 0.7	8.6 ± 0.30	8.7 ± 2.24	9.9 ± 0.16	6.0 ± 0.32	4.3 ± 0.38
Quinacrine at 75 mg/kg	24.9 ± 0.69	24.0 ± 0.55	−1.0 ± 0.24	8.0 ± 0.12	8.3 ± 1.08	10.1 ± 0.25	4.6 ± 0.59	2.8 ± 0.48
AG at 25 mg/kg	22.7 ± 0.45	23.2 ± 0.85	0.5 ± 0.55	7.6 ± 0.31	14.6 ± 2.63	10.2 ± 0.15	9.1 ± 0.59	7.6 ± 0.40
AG at 50 mg/kg	24.6 ± 0.22	25.3 ± 0.32	0.8 ± 0.3	7.7 ± 0.21	13.8 ± 2.31	10.0 ± 0.17	8.1 ± 0.44	6.3 ± 0.35
AG at 75 mg/kg	24.7 ± 0.68	25.3 ± 0.86	0.6 ± 0.44	8.2 ± 0.6	10.8 ± 1.71	9.4 ± 0.19	9.1 ± 0.50	7.3 ± 0.51
HAG at 25 mg/kg	24.2 ± 0.40	23.5 ± 0.84	−0.7 ± 0.81	6.9 ± 0.16	17.1 ± 1.79	9.7 ± 0.29	7.7 ± 0.77	5.9 ± 0.75
HAG at 50 mg/kg	24.9 ± 0.66	24.9 ± 0.64	0 ± 0.24	8.2 ± 0.37	13.5 ± 1.71	9.9 ± 0.21	6.8 ± 0.52	5.1 ± 0.37
HAG at 75 mg/kg	23.6 ± 0.44	24.0 ± 0.52	0.4 ± 0.39	7.5 ± 0.19	8.3 ± 1.19	9.8 ± 0.29	7.7 ± 0.60	6.4 ± 0.59
Resveratrol at 25 mg/kg	23.8 ± 0.49	24.0 ± 0.47	0.2 ± 0.34	7.4 ± 0.21	15.3 ± 1.89	9.7 ± 0.17	7.5 ± 0.22	6.4 ± 0.17
Resveratrol at 50 mg/kg	23.9 ± 0.42	24.3 ± 0.39	0.4 ± 0.61	7.2 ± 0.18	8.7 ± 0.97	9.1 ± 0.64	5.5 ± 0.49	4.5 ± 0.42
Resveratrol at 75 mg/kg	23.8 ± 0.32	24.4 ± 0.39	0.6 ± 0.31	7.5 ± 0.28	7.2 ± 1.24	9.9 ± 0.24	6.7 ± 0.67	5.5 ± 0.64

Values are group averages ± SE.

WBC, white blood cells. RBC, Red Blood Cells.

† Millions per cubic milliliter of blood.

†† Billions per cubic milliliter of blood.

As expected, all tested agents have successfully suppressed the progression of colitis in a dose-dependent manner, *ranging in the following order from the most to the least effective: Resveratrol > Quinacrine > HAG > BB-Cl-Amidine > AG > Cl-Amidine > Olsalazine* + *Cyclosporine A combination > 5-ASA.* In general, the highest tested doses of each compound were most effective against colitis, reducing moderate to severe inflammation and mild ulceration in the DSS only treated group with a histological score of 25.9 ± 1.6 to mostly mild inflammation and ulceration in the drug-treated groups with histological scores in the range of 7.2 ± 1.2 for the Resveratrol group at 75 mg/kg group to 19.4 ± 2.5 for the 5-ASA group at 25 mg/kg group. Overall, all tested CAM and small molecule compounds were up to two fold more efficient in inhibiting the severity and extent of colitis in comparison to the clinically used FDA-approved agents 5-ASA and Olsalazine / Cyclosporine A combinations.

**Figure 2:**
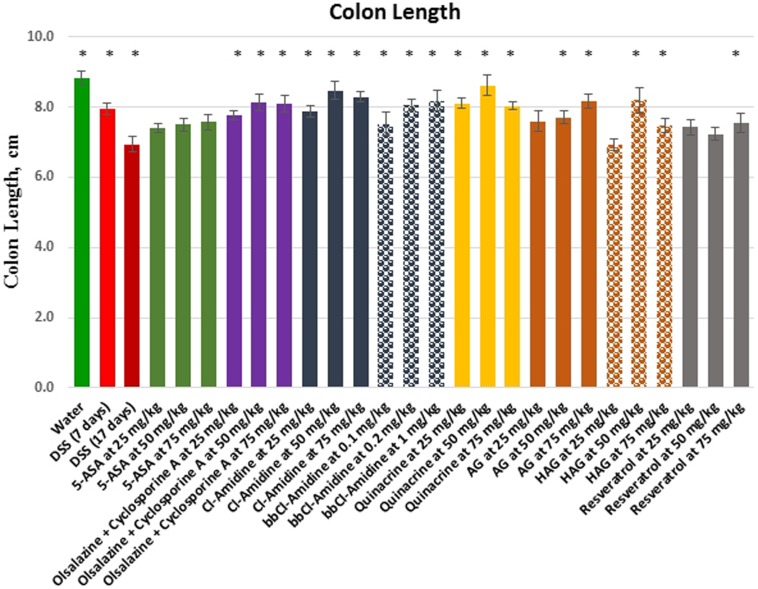
Effects of the treatment on colon length in the DSS model of colitis Values represent mean ± S.E. * indicates significant difference (*p* < 0.05) from 17 days DSS only group.

Since mouse colon length decreases with inflammatory stress, and ulceration, we also used this parameter as an indicator of inflammation severity (Figure 2, Table 1). The average colon length of the water control group was 8.8 ± 0.2 cm. DSS treatment caused colon shrinkage to 7.9 ± 0.2 cm on average at day 7, and further to 6.9 ± 0.20 cm at the 17 day time point. In contrast, the average colon length for all tested drugs at the day 17 time point varied from 7.4 ± 0.1 cm for the lowest used concentration of 5-ASA to 8.6 ± 0.3 cm for the middle concentration of quinacrine, which is within the range of colons lengths found in healthy control animals. The compounds ranged in the following order *from the most to the least effective in preventive colon shrinkage: Quinacrine > Cl-Amidine > HAG > BB-Cl-Amidine > AG > Olsalazine* + *Cyclosporine A combination > 5-ASA > Resveratrol.* In general, all tested compounds beat current FDA-approved treatment strategies in their efficacy. These results are consistent with histological inflammatory scores for the tested groups. Surprisingly, animals treated with resveratrol, which demonstrated the lowest immunological scores, had relatively shorter colons, but the colons were *still significantly longer, than those of DSS only treated animals*. It is also worth mentioning that the middle doses of most tested compounds were more effective than both lower and higher doses used, in regard to colon shrinkage prevention.

**Figure 3:**
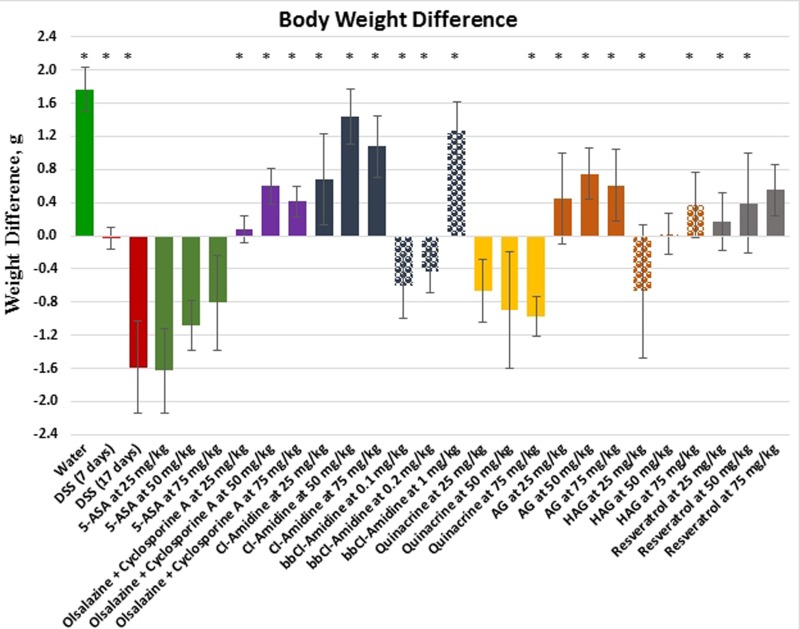
Body weight difference after the treatment in the DSS model of colitis Values represent mean ± S.E. * indicates significant difference (*p* < 0.05) from 17 days DSS only group.

An additional parameter – difference in animal body weight on day 17 (last experimental day) in comparison to the day 0 (experiment starting day) – was used to assess the overall health of the mice at the end of experiment (Figure 3, Table 1), as well as potential toxicity of the tested drugs. On average, the water control group of healthy animals gained 1.8 g of body weight during the course of experiment, whereas the DSS only treated group lost almost the same amount of body weight (−1.6 g). The FDA-approved anti-colitis drug 5-ASA demonstrated comparable weight loss to the DSS only group, ranging from 0.8g to 1.6g depending on the dose used, whereas animals treated with another FDA-approved combination of Olsalazine + Cyclosporine A showed a moderate weight increase of 0.1 – 0.6 g depending on the doses used. The weight change dynamic was markedly different for all tested compounds. While animals treated with the small molecule compound Cl-Amidine gained weight comparable to the water group (0.7 – 1.4 g) at all used doses, its derivative, BB-Cl-Amidine, caused weight loss at lower and middle doses, but one of the largest weight gains for the highest used dose – 1.3 g for the 1 mg/kg dose. CAM compounds were also varied in this regard. AG caused a substantial dose-dependent weight gain of 0.5 – 0.8 g, while only the highest dose of HAG allowed the animals to gain weight. Resveratrol caused a moderate dose-dependent body weight increase of 0.2 – 0.6g, similar to the AG and Olsalazine + Cyclosporine A groups. The overall order of the compounds from the most to the least effective in regards of helping to maintain body weight and overall well-being was as follows: *Cl-Amidine > BB-Cl-Amidine > AG > Resveratrol > Olsalazine + Cyclosporine A > HAG > Quinacrine > 5-ASA.* Once again, CAM and small molecule compounds tested here prevail over current FDA-approved colitis therapeutics.

### Inflammatory stress is reduced in treated mice

We have previously shown that all compounds tested here can be used to prevent and treat mouse colitis [14–16, 18, 29, 41–45] through, at least partially, the induction of inflammatory cell apoptosis mechanism [14, 42].

**Figure 4:**
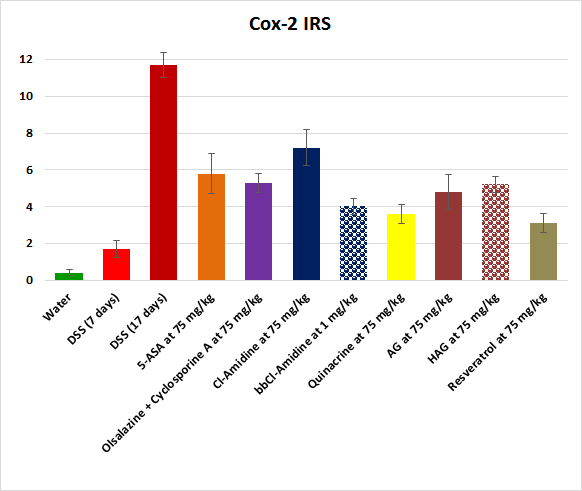
Effects of the treatment on the colon Cox-2 immunoreactivity score in the DSS model of colitis Values represent the average scores for each group ± S.E. (*N* = 10 per group). The differences between individual experimental groups and 17 days DSS only group were statistically significant (*p* < 0.05) for all groups.

To further compare the efficacy of tested compounds, we examined the expression of an inflammatory marker, COX-2, using IHC. Since the histological inflammation scores showed unconditional dose dependence for all tested compounds, only the colons of animals treated with the highest doses of each compound were used for IHC. Figure 4 demonstrates the quantification results of IHC staining with COX-2. Images of representative colon sections are shown in Supplementary Picture 1. Overall, COX-2 levels were elevated in the DSS only treated mice, and were statistically significantly reduced in all treatment groups in the following order, complementing histopathology results: *Resveratrol > Quinacrine > BB-Cl-Amidine > AG > HAG > Olsalazine + Cyclosporine A > 5-ASA > Cl-Amidine*.

Similarly, the total white blood cell (WBC) count and lymphocyte count in particular, reflecting systemic inflammation load, was increased in DSS only treated groups, but significantly reduced in the treatment groups (Table 1). There was no significant difference in red blood cell (RBC) count for all groups, thereby excluding any anemia-like conditions in the treatment groups. Overall, these results are consistent with the notion that all used treatment agents and conditions suppress DSS-induced colitis in a dose dependent manner.

## DISCUSSION

The objective of this study was to rank eight treatments in regard to their anti-colitis efficiency in a DSS mouse model. In order to do so, we quantitatively assessed four endpoints – histological inflammation scores, colon lengths, body weight changes, and immunoreactivity scores for the COX-2 marker of inflammation. Also we demonstrate a very similar ranking order for the tested compounds, although there were some serious variations in this order in regards to each endpoint. For instance, quinacrine at the dose of 75 mg/kg was ranked #1 or #2 in all but one category – body weight loss, in which it was one of the worst compounds. This type of rank differences prevented us from drawing a direct conclusion about the potency of each compound in the treatment of colitis. Therefore we have developed a novel tool – composite scoring – that combines all measured endpoint parameters.

We first ranked each of the endpoint measurements in order of primary importance (levels of inflammation and the COX-2 IRS) and secondary, but nonetheless also of *vital* importance (colon length and body weight difference). This final composite score primarily relies on the two primary endpoint measurements. However, if the secondary measurements are highly different from what is expected under the water only control group, which is considered the golden health standard, this will increase the composite score. Two tuning parameters are introduced: the first controls the threshold of how different the secondary scores need to be from the water group before contributing to the composite score, and the second is the magnitude of the penalty that is levied from these secondary endpoints. These tuning parameters are then chosen such that the treatment groups are most different from each other, measured by the F-statistic arising from the one-way analysis of variance of the composite scores. On the basis of these approaches and using a multiple comparisons procedure with a Benjamini-Hochberg false discovery rate correction, the eleven treatment protocols are ranked and grouped as indicated below, with those in the same group not significantly different from each other at 5% level of significance. Details of the statistical analysis approach leading to the final ranking provided here will be published separately.

Using this approach, we were able to rank all tested compounds based on composite score in the following order:

Resveratrol > BB-Cl-Amidine ≥ Quinacrine > HAG > AG > Cl-Amidine > Olsalazine + Cyclosporine A > 5-ASA.

Overall, all tested CAM and small molecule compounds were more effective against colitis than current FDA-approved anti-colitis treatments, with resveratrol on top of the list and 5-ASA at the bottom. These results are consistent with our conclusions drawn for each endpoint. However there are some interesting exceptions. Quinacrine, which was ranked as one of the top two compounds in regard to each endpoint, except for body weight change, was ranked #3 overall, surprisingly losing its place to BB-Cl-Amidine that demonstrated rather mediocre results in regard to each endpoint.

The results of this study provide a useful multi-comparisons procedure composite score to assess biological potency of different compounds, as well as pre-clinical data for the movement of specific treatments examined here to clinical trials.

## MATERIALS AND METHODS

### Chemicals and reagents

Olsalazine sodium was purchased from Selleckchem (USA), 5-Aminosalicylic acid (5-ASA) and resveratrol – from Acros Organics (USA), and Cyclosporine A – from Santa Cruz Biotech (USA). Quinacrine dihydrochloride was obtained from Sigma (USA), and dextran sulfate sodium (molecular weight, 36,000-50,000) was purchased from Advanced Technology & Industrial Co., Ltd. (Hong Kong).

The synthesis of Cl-amidine has been described previously [20, 46], as well as its modified version, BB-Cl-Amidine [23]. The American Ginseng (AG) *Panax quinquefolius* extract has been described previously in detail by our laboratory [14], as well, as we have recently described the generation of the Hexane fraction of AG (HAG) [44].

### Animals and DSS mouse model of colitis

Male C57BL/6 mice, 12 weeks of age, weighing 20 to 29 g were obtained from The Jackson Laboratories (Bar Harbor, ME). All mice were kept in clean, dedicated animal quarters and provided food and water. Care and use of animals was overseen by the Animal Resource Facility (ARF) of the University of South Carolina under the direction of a veterinarian. The ARF is fully accredited by the Association for Assessment and Accreditation of Laboratory Animal Care International, is registered with the U.S. Department of Agriculture (56-R-003) and has an active letter of Assurance of Compliance on file at the NIH. The Institutional Animal Care and Use Committee (IACUC) of the University of South Carolina approved this study.

The DSS mouse model of colitis used here is similar to the one used previously by our lab [29, 40]. Animals received either water or 1.5% DSS dissolved in water for 7 days. Seven days after the initial DSS treatment, we sacrificed 10 animals to monitor colitis progression, and for the rest of the animals (10 mice per group) initiated a daily oral administration of the following agents:

Vehicle solution

5-ASA at 25, 50, or 75 mg/kg;

Olsalazine at 100 mg/kg in combination with 25, 50, or 75 mg/kg of Cyclosporin A;

Cl-Amidine at 25, 50, or 75 mg/kg;

BB-Cl-Amidine at 0.1, 0.2, or 1 mg/kg;

Quinacrine at 25, 50, or 75 mg/kg;

AG at 25, 50, or 75 mg/kg;

HAG at 25, 50, or 75 mg/kg;

Resveratrol at 25, 50, or 75 mg/kg.

All agents were administered by oral gavage once daily, except for Cl-Amidine and BB-Cl-Amidine, which were dissolved in the drinking water and available to the mice *ad libitum*. 1.5% DSS treatment continued in the indicated groups. Water group of animals did not receive any DSS, nor treatment compounds. Control animals have received a vehicle solution as a treatment.

The doses of all agents were chosen based on the following criteria:

- being in the range taken by humans;
- being non-toxic; and
- known to suppress colitis in mice based on previously published studies [14–16, 18, 29, 41–45].

Following 10 days of treatment with the above indicated compounds, on day 17 the mice were sacrificed and the colons were harvested for further processing and analysis. Blood was collected prior to the sacrifice. Colons were transected longitudinally, pinned open, and rinsed with PBS. Colon lengths were recorded, and colons were processed for hematoxylin-eosin (H&E) staining and immunohistochemistry by fixing in formalin overnight, then Swiss-rolling and embedding in paraffin.

### Quantification of inflammation

Sectioned colon samples were stained with H&E. The sections were microscopically examined for histopathological changes using the following scoring system. Histology score was determined by two blinded investigators (AC and EW) as a product of multiplication for each of the three histological features by the percent area of involvement [42, 47]. Inflammation severity was scored as 0 for none, 1 for minimal, 2 for moderate, and 3 for severe; inflammation extent as 0 for none, 1 for mucosa, 2 for mucosa and submucosa, and 3 for transmural; crypt damage as 0 for none, 1 for one-third of crypt damaged, 2 for two-thirds of crypt damaged, 3 for crypt loss and surface epithelium intact, 4 for crypt loss and surface epithelium loss; and percent area involvement was scored as 0 for 0%, 1 for 1–25%, 2 for 26–50%, 3 for 51–75%, and 4 for 76–100%. Therefore, the minimum score is 0, and the maximum score is 40.

### Immunohistochemical staining

For immunohistochemical staining, formalin-fixed, paraffin-embedded serial sections of mouse colon tissues were incubated overnight with antibodies against Cox-2 (polyclonal, 1:2000 dilution; Cayman Chemical) by slow rocking using the Antibody Amplifier (ProHisto, Columbia, SC) to ensure even staining and reproducible results. After incubation with primary antibodies, sections were processed using EnVision+ System-HRP kits (DakoCytomation, Carpinteria, CA) according to kit protocols. The chromogen was diaminobenzidine, and sections were counterstained with 1% methyl green. Intensity and degree of staining were evaluated independently by three blinded investigators (AC, AC and EW). For each tissue section, the percentage of positive cells was scored on a scale of 0–5 for the percentage of tissue stained: 0 (0% positive cells), 1 (<10%), 2 (11–25%), 3 (26–50%), 4 (51–80%), or 5 (>80%). Staining intensity was scored on a scale of 0–3: 0 (negative staining), 1 (weak staining), 2 (moderate staining), or 3 (strong staining). The two scores were multiplied, resulting in an immunoreactivity score (IRS) value ranging from 0 to 15.

### Statistics

Mean differences between groups were compared by one-way ANOVA with Scheffé's multiple comparison tests. A Pearson correlation coefficient was applied for comparisons of the trends. *P* ≤ 0.05 was chosen for significance.

A statistical analysis that allowed final ranking of the compounds' overall anti-colitis effectiveness based on a composite score was done using a multiple comparisons procedure with a Benjamini-Hochberg false discovery rate correction which will be fully described and discussed in a separate paper.

## SUPPLEMENTARY MATERIAL



## Abbreviations

5-ASA: 5-aminosalicylic acid
AG: American Ginseng
CAM: complementary and alternative medicine
DSS: dextran sulfate sodium
HAG: hexane fraction of AG
H&E: hematoxylin and eosin
IBD: inflammatory bowel disease
IHC: immunohistochemistry
IRS: immunoreactivity score
PAD: protein arginine deiminase
UC: ulcerative colitis.
