# Supplementary material for: Looking for the best anti-colitis medicine: A comparative analysis of current and prospective compounds
Source: Oncotarget. 2016 Dec 10;8(1):228–37. doi: 10.18632/oncotarget.13894 (PMC5352114; doi:10.18632/oncotarget.13894)
Supplement: Supplementary file 1 [file oncotarget-08-228-s001.pdf]

Looking for the best anti-colitis medicine: A comparative analysis of current and prospective compounds

Supplementary Material

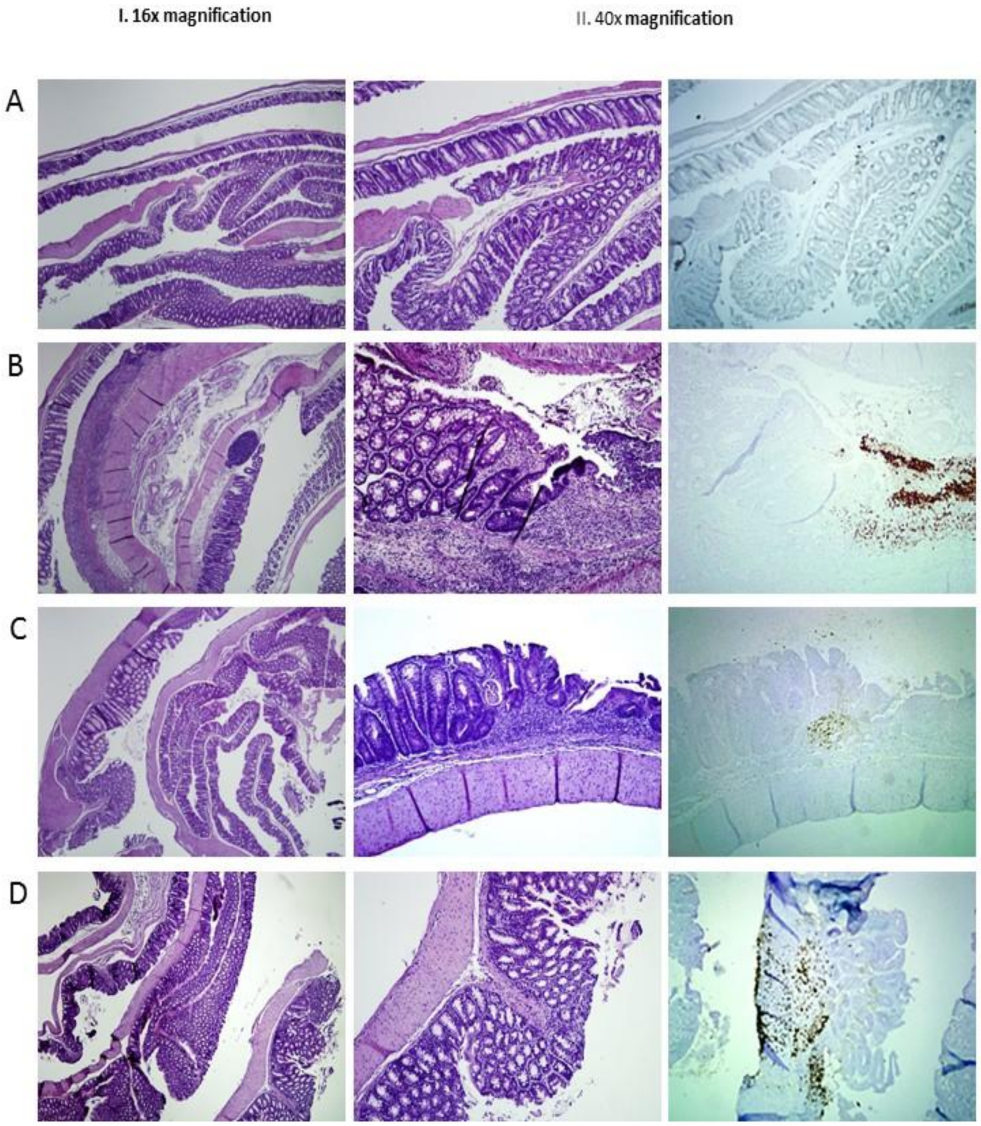

Supplemental Figure 1. Representative histological (H&E stained) and IHC (Cox-2 stained) sections from the treated groups. A. Water; B. 2% DSS; C. 1.5% DSS + 50 mg/kg Quinacrine; D. 1.5% DSS + 1mg/kg BB-Cl-Amidine. Consecutive sections of the corresponding colons were used for H&E and Cox-2 staining.
